# Mitochondrial Dysfunction and Heart Disease: Critical Appraisal of an Overlooked Association

**DOI:** 10.3390/ijms22020614

**Published:** 2021-01-09

**Authors:** Giandomenico Bisaccia, Fabrizio Ricci, Sabina Gallina, Angela Di Baldassarre, Barbara Ghinassi

**Affiliations:** 1 MIUR Department of Excellence, Department of Neuroscience, Imaging and Clinical Sciences, University “G.d’Annunzio” of Chieti-Pescara, Via Luigi Polacchi, 11-66100 Chieti, Italy; giandomenico.bisaccia@studenti.unich.it (G.B.); sabina.gallina@unich.it (S.G.); 2 Department of Clinical Sciences, Lund University, E-205 02 Malmö, Sweden; 3 Casa di Cura Villa Serena, Città Sant’Angelo, 65013 Pescara, Italy; 4 Department of Medicine and Aging Sciences, University “G.d’Annunzio” of Chieti-Pescara, 66100 Chieti, Italy; angela.dibaldassarre@unich.it (A.D.B.); b.ghinassi@unich.it (B.G.)

**Keywords:** mitochondria, heart failure, mitochondrial dynamics, cardiomyopathy, cardiac energetics

## Abstract

The myocardium is among the most energy-consuming tissues in the body, burning from 6 to 30 kg of ATP per day within the mitochondria, the so-called powerhouse of the cardiomyocyte. Although mitochondrial genetic disorders account for a small portion of cardiomyopathies, mitochondrial dysfunction is commonly involved in a broad spectrum of heart diseases, and it has been implicated in the development of heart failure via maladaptive circuits producing and perpetuating mitochondrial stress and energy starvation. In this bench-to-bedside review, we aimed to (i) describe the key functions of the mitochondria within the myocardium, including their role in ischemia/reperfusion injury and intracellular calcium homeostasis; (ii) examine the contribution of mitochondrial dysfunction to multiple cardiac disease phenotypes and their transition to heart failure; and (iii) discuss the rationale and current evidence for targeting mitochondrial function for the treatment of heart failure, including via sodium-glucose cotransporter 2 inhibitors.

## 1. Introduction

The normal heart consumes on average between 6 and 30 kg of ATP per day [1]. Over 95% of the ATP required for proper cardiac functioning is produced within the mitochondria, which are generally known as the cells’ powerhouses. Basic, clinical, and translational research on cardiac mitochondria has received much interest in the last decades. 

Mitochondrial disorders are considered among the most frequent genetic diseases [2] and are associated with a high incidence of cardiac involvement [3] featuring myocardial metabolic disturbances with ensuing left ventricular dysfunction and conduction disorders. Nevertheless, the burden of inherited heart muscle disease linked to mitochondrial dysfunction is relatively low compared with acquired cardiomyopathies, such as ischemic heart disease and diabetic cardiomyopathy [4], where mitochondrial dysfunction plays a significant pathophysiological role and leads the transition from the normal heart to the final common pathway of cardiac disease, specifically heart failure. 

In the current bench-to-bedside review, (i) we examine the main functions of mitochondria in the heart, including energy production, cell growth, calcium transport, apoptosis, and the handling of free radicals; moreover, (ii) we discuss the mechanisms of mitochondrial (dys)function underlying acquired heart disease, and (iii) we explore the most recent advances in the field of mitochondrial-targeted therapies directed to restore an optimal metabolic milieu for the treatment of heart failure [5].

## 2. Mitochondrial Structure and Function in the Normal Heart

### 2.1. Origin and Morphology of Mitochondria

It is widely accepted that mitochondria are derived from bacteria that, billions of years ago, lived inside eukaryotic cells [6]. The *endosymbiotic theory* states that such bacteria gave early eukaryotes the capability to perform oxidative phosphorylation. Thus, they became pivotal to the very existence of eukaryotes and finally were embedded in the eukaryotic cellular structure. Of the >1000 genes constituting the human mitochondrial genome, only 37 are encoded in the mitochondrial DNA, the majority of genes being encoded in the nuclear DNA [7]. Mitochondria have a double-membraned organization, consisting of an outer membrane and an inner membrane delimiting the intermembrane space; infoldings of the inner membrane constitute the cristae, while the space delimited by the inner membrane is termed the matrix.

The outer membrane is very similar to other cell membranes, with a 1:1 protein-to-lipid ratio; the most represented protein in the outer membrane is porin, an integral membrane protein facilitating inflow and outflow of small molecules [8]. 

The inner membrane is home to the enzymatic machinery that performs oxidative phosphorylation. This machinery is composed of four protein complexes and ATP synthase [9]. Specific to the mitochondrial inner membrane is the negatively charged phospholipid cardiolipin, a key component for the functioning of enzyme complexes [10].

The mitochondrial matrix contains mitochondrial DNA, ribosomes, RNAs, and enzymes for the oxidation of pyruvate and fatty acids. Inside the matrix, the Krebs cycle takes place. 

The morphology and functioning of cardiac mitochondria vary based on the physiological milieu, stage of development, and disease. Moreover, heterogeneity in mitochondrial morphology and position inside the cardiomyocyte likely reflects the existence of different patterns of mitochondrial response to physiological and pathological stimuli [11].

### 2.2. Mitochondrial Networks in the Heart: Biogenesis, Mitophagy, and Mitochondrial Dynamics

The proper functioning of cardiomyocytes requires continuous harmonization of mitochondrial function. This is obtained via the adaptation of mitochondria to current energy needs, which require dynamic expansion and contraction of mitochondrial pools, development of new mitochondria, and removal of “old” organelles; these processes, known as fusion, fission, biogenesis, and mitophagy [12], respectively, are key to the genesis and dynamics of mitochondrial networks.

Mitochondrial fusion and fission allow mitochondria to exchange components and ensure a proper myocellular distribution of these organelles, building a complex network of interactions commonly referred to as mitochondrial dynamics [13]. 

Mitochondrial fusion consists of the merging of, respectively, the outer and inner mitochondrial membranes of different mitochondria. This process is mediated by specific proteins, including mitofusins 1 and 2 (MFN1 and MFN2) on the outer membrane, and optic atrophy 1 (OPA1) on the inner mitochondrial membrane and intermembrane space [13].

Mitochondrial fission allows for redistribution of mitochondria inside the cardiomyocyte. It requires interaction of a cytosolic protein, namely dynamin-related protein 1 (DRP1), with an outer membrane protein called mitochondrial fission 1 protein (FIS1) [13], in forming the mitochondrial fission complex. 

Mitochondrial dynamics is the result of continuous balancing between fusion and fission processes. For example, disrupting the fusion machinery determines mitochondrial fragmentation, inevitably leading to apoptosis [14]. Other proposed roles for fusion and fission have been hypothesized in various mitochondrial processes, including mitochondrial DNA (mtDNA) deletion and bioenergetics, and in different cardiac diseases, including cardiomyopathies and heart failure [13].

Mitochondrial biogenesis is the process by which mitochondria grow up and multiply [15]. In muscle cells, including cardiomyocytes, the division of pre-existing mitochondria is triggered by physical stress and various chemical signals. The expansion of the cardiac mitochondrial pool is needed in order to maintain a production of ATP sufficient for cardiac contractility. Biogenesis occurs via a transcriptional cascade involving, among others, the activation of PPARγ coactivator 1α (PGC-1α) and a subsequent increase in nuclear respiratory factors (NRFs), which cause the expression of mitochondrial DNA and proteins. Notably, the activation of PGC-1α also causes an increase in cell respiration and production of ATP [15].

Mitophagy is an autophagy process by which mitochondria are degraded by lysosomes for preserving mitochondrial homeostasis [16]. Mitophagy can be considered a “quality check” process that prevents accumulation of dysfunctional mitochondria, an event that would lead to activation of inflammatory pathways and cell death [17]. Triggers of mitophagy in the heart comprise hypoxia and excessive ROS production (i.e., during reperfusion). 

The dysregulation of mitochondrial dynamics and mitophagy found is associated with defective removal of damaged mitochondria and subsequent activation of inflammatory responses, paving the way to cardiomyocyte aging [18] and heart failure (HF) [19]. In particular, disruption of quality control mechanisms may allow a malfunctioning mitochondrion to propagate its defect to the cell-level, ultimately leading to ROS overproduction and apoptosis [20]. 

Antioxidant systems (superoxide dismutase, glutathione peroxidase, glutathione reductase, etc.) represent the first level of quality control, preventing molecular damage from happening inside mitochondria [21]. When antioxidant systems fail, repair processes (molecular chaperones, mtDNA repair complexes, reductase systems) take over to recover the molecule if possible. If a molecule is irreparably damaged, an intramitochondrial proteolytic system performs its clearance. Mitochondrial biogenesis, mitophagy, and mitochondrial dynamics play an active role in this context, so that an imbalance between fission and fusion events, as well as increased mitophagy and reduced biogenesis, potentially lead to diffuse cell damage and death [20], activation of proinflammatory pathways and, at the organism-level, aging and HF [21].

### 2.3. Mitochondrial Bioenergetics and Ion Handling in the Heart

Production of reduced equivalents, nicotinamide adenine dinucleotide (NADH) and flavin adenine dinucleotide (FADH_2_), is of utmost importance for appropriate mitochondrial functioning. Such products are obtained by metabolization of different substrates—mainly free fatty acids within the myocardium—converging on the Krebs cycle to fuel the oxidative phosphorylation process [22]. ROS, previously considered by-products of oxidative phosphorylation, are now being seen as important signaling molecules [23].

Another emerging feature of cardiac mitochondrial function is calcium handling [24,25]. In physiological conditions, intracellular calcium is stored in the sarcoplasmic reticulum, and calcium transients to the cytosol are sensed by nearby mitochondria, triggering a burst in oxidative phosphorylation. 

The outer mitochondrial membrane shows high permeability to calcium, so that cytosol and intermembrane space calcium concentrations are virtually equal. Calcium enters the matrix via the mitochondrial calcium uniporter (MCU) [26,27] complex on the inner mitochondrial membrane, which shows adaptive response patterns to low-versus-high calcium concentrations [28]; during diastole, the MCU does not import calcium due to low cytosol concentrations and subsequent blockage by MCU regulatory proteins. Instead, during systole, rising calcium concentrations trigger a conformational change of said proteins, and calcium import is allowed.

Mitochondrial calcium export, on the other hand, requires the functioning of the Li^+^-permeable Na^+^–Ca^2+^ exchanger (NCLX), a member of the Na^+^–Ca^2+^ exchanger family of antiporters. NCLX may maintain a steady state in mitochondrial calcium content by exporting the same quantity of calcium imported by the MCU. MCU and NCLX functioning is regulated by different means, including phosphorylation and variations in the mitochondrial membrane potential. 

In conditions of calcium overload, NCLX exporting capacity is overcome, and calcium content rapidly increases in the mitochondrial matrix. This triggers the aggregation and opening of the mitochondrial permeability transition pore (MPTP) on the inner mitochondrial membrane, causing mitochondrial content to be released into the cytoplasm. This leads to a staggering loss in mitochondrial membrane potential, rapid ATP deprivation, and finally cell death.

In order to investigate the specific role of MCU in mitochondrial calcium handling, an MCU knockout mouse model was first developed [29] and showed altered calcium loading capacity but no specific cardiac alterations. The same finding was confirmed in a follow-up study [30]. Nevertheless, subsequent investigations demonstrated that acute—but not chronic—deletion of MCU confers protection against cell death in ischemia/reperfusion injury (IRI) mouse models [31] by reducing MPTP activation. Interestingly, MCU was also found to be essential for the fight-or-flight adrenergic response [32].

## 3. Mitochondrial Dysfunction and Cardiovascular Disorders

**Figure 1:**
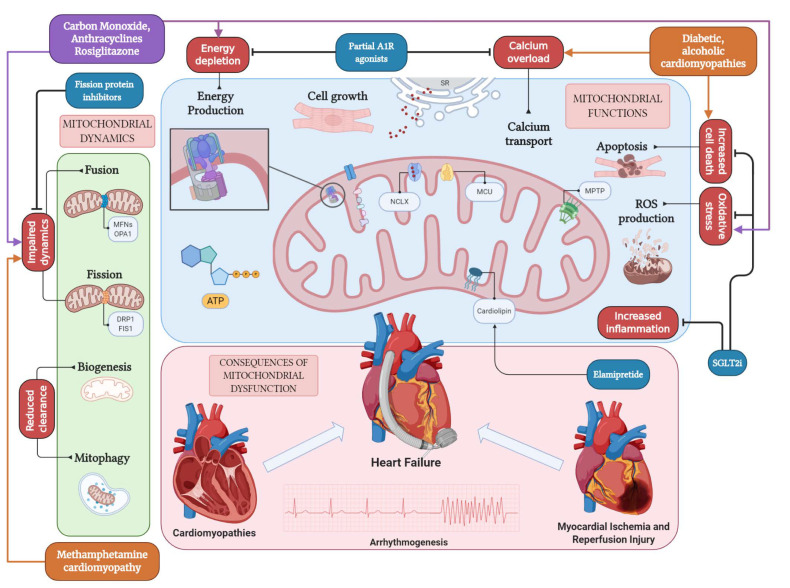
**Pathways of mitochondrial dysfunction leading to cardiovascular disease.** Mitochondria serve the cardiomyocyte by performing numerous functions, including energy production, cell growth, calcium transport from and to the sarcoplasmic reticulum (SR, in figure), regulation of cell death, and production of ROS. Mitochondrial dynamics through fission and fusion cycles, as well as biogenesis and mitophagy, allow for the regulation of the mitochondrial pool. Many of these functions are halted by toxins and disease. Diabetes and alcohol abuse are linked to calcium overload and increased cell death; methamphetamine cardiomyopathy determines a halt in the regulation of mitochondrial dynamics; exposure to carbon monoxide and administration of anthracyclines and rosiglitazone cause energy depletion, calcium overload, and an increase in oxidative stress. Consequences of mitochondrial dysfunction in the heart include myocardial infarction and ischemia/reperfusion injury, cardiomyopathies, and arrhythmias; finally, mitochondrial abnormalities have been described in the pathogenesis of heart failure. Created with BioRender.com.

Mitochondrial dysfunction is particularly sensitive to cells with high levels of energy consumption, i.e., cardiomyocytes [6]. In the following sections, specific relationships between mitochondrial dysfunction and different cardiovascular (CV) disorders are discussed (Figure 1).

### 3.1. Myocardial Infarction and Ischemia/Reperfusion Injury

Mitochondrial dysfunction plays a pivotal role in myocardial infarction (MI) and ischemia/reperfusion injury (IRI); indeed, factors modifying mitochondrial behavior (i.e., age, sex, statin use, and comorbidities like diabetes and metabolic syndrome) directly impact the efficacy of cardioprotection strategies [33].

At the onset of myocardial infarction, oxygen and nutrient deprivation cause the cardiomyocytes to shift from oxidative phosphorylation to anaerobic glycolysis. This results in the lowering of the intracellular pH to levels <7.0 [34]. The rising intracellular concentration of protons determines activation of the Na^+^/H^+^ ion exchanger, leading to an increasing Na+ concentration in the cytoplasm. This triggers an inversion in the functioning of the Na+/Ca+ ion exchanger, which determines intracellular (later, mitochondrial) calcium overload. 

When reperfusion begins, pH in the cardiomyocyte returns to normal levels, and rising oxygen concentrations lead the mitochondrial machinery to a burst in ROS production and further mitochondrial calcium overload (mitochondrial re-energization); these events determine the opening of the MPTP, with release of mitochondrial content in the cytoplasm [34] and impairment of mitochondrial fission [35]. At this point, the entity of mitochondrial leak will determine the cardiomyocyte’s survival or death; if the leak is minimal, the cell recovers and its functioning is restored; if the leak is major, the cardiomyocyte dies. 

Interestingly, a large conductance, calcium-activated K^+^ channel (BK_Ca_), expressed on the plasma membrane of most cell types but characteristically found on the inner mitochondrial membrane of adult cardiomyocytes (mitoBK_Ca_), has been shown to confer protection from IRI by increasing the mitochondrial calcium capacity and reducing ROS production [36]. Increased ROS production during preconditioning triggers the opening of the mitoBK_Ca_ channel, leading to a K^+^ influx in mitochondria with an ensuing reduction in Ca^2+^ influx and internal membrane depolarization, overall preventing calcium overload, MPTP opening, and cardiomyocyte death.

Another possible consequence of IRI is the impairment of mitochondrial membrane potential, which, as discussed below (paragraph 3.4 Conduction disorders) may determine the onset of arrhythmias during recovery from MI [37].

The prevention of IRI via administration of antioxidants has been studied in both experimental and clinical settings [38] with unconvincing results; a potential explanation for this is that antioxidants fail to reach the cardiomyocyte. Other proposed therapeutic targets include mitochondrial proteins involved in fusion and fission, and the mitoBK_Ca_ channel. Experimental studies showed promising results as administration of fission proteins inhibitors reduced MI size and improved mitochondrial function in rodents [39], but more recent data did not confirm such findings and point to possible inhibitor-related mitochondrial damage [40]. Similarly, results from studies exploring the therapeutic benefits of mitoBK_Ca_ channel activation are on the rise; mitoBK_Ca_ agonists include both synthetic substances (NS1619, NS11021 [41]) and many endogenous molecules, including nitric oxide, carbon monoxide (CO), and hydrogen sulfide [36].

The efficacy of specific cardioprotection strategies has been showed to directly depend on the mitochondrial machinery; in particular, in the setting of ischemic preconditioning of the cardiomyocyte, ischemia-induced decrease in ATP production leads to activation of mitochondrial ATP-sensitive K^+^ channels and a subsequent increase in K^+^ influx. This would trigger mitochondria to release ROS, activating mediators of cardioprotection that prevent the opening of the MPTP at reperfusion, thus limiting IRI [33]. With regard to ischemic postconditioning, the mechanistic pathways implicated converge on the mitochondria as well, with prevention of MPTP opening by activation of cardioprotective pathways [33].

### 3.2. Drug-Induced and Toxic Cardiomyopathies

Drug-induced cardiomyopathy (CMP) embeds a subgroup of dilated CMPs featuring alterations in cardiac structure and function induced by administration of toxins and medications. Recognized etiologic agents include ethanol, cocaine and other illicit drugs, and anticancer drugs including anthracyclines, imatinib, and trastuzumab. Toxic CMP, on the other hand, requires exposure to poisonous substances like carbon monoxide and arsenic [42].

#### 3.2.1. Alcohol

“Alcoholic” CMP is defined as cardiomyopathy caused by ethanol intoxication in patients with histories of alcohol abuse. It has been recognized as an entity [43], even though a definitive elucidation on its specific pathophysiology is still needed. 

Proposed mechanisms of ethanol-mediated cardiac damage include electromechanical uncoupling, disruption in calcium homeostasis, increases in oxidative stress, and suppression of mitochondrial function, resulting in increased apoptosis [44]. Recently, a mouse model has shown that chronic ethanol consumption promotes mitochondrial fragmentation and dysfunction, directly leading to cell death [45]. 

#### 3.2.2. Methamphetamine

Methamphetamine (METH) is an illicit drug, with toxic effects for the nervous and CV system, characterized by high addictiveness and associated with arrhythmias and cardiomyopathy [46]. While the pathophysiology behind the neurological effects of METH are well understood, little knowledge is available with regard to its cardiotoxicity. However, recent studies have shown that a specific fingerprint can be traced for METH-related CMP, which is characterized by fibrotic remodeling of the left ventricle in humans, potentially leading to heart failure [47]. 

METH-induced cardiac contractile dysfunction can be explained by mitochondrial abnormalities; indeed, in a mouse model, METH exposure led to impaired OXPHOS and a decrease in the expression of mitochondrial protein FIS1, which determines an excess in fusion events [47]. METH-induced impairment in mitochondrial dynamics may require METH binding the SigmaR1 modulator, so that pharmacological prevention of this binding may represent a therapeutic target for METH-induced CMP.

#### 3.2.3. Anticancer Drugs

Among anticancer drugs, anthracyclines (ANTs) have garnered much attention as powerful cancer agents with dangerous cardiotoxic effects. However, the anticancer effects of ANTs (mainly due to inhibition of topoisomerase 2) do not fully explain their cardiotoxicity. One of the most accepted explanations for anthracycline cardiotoxicity is an increase in cardiomyocyte ROS production, due to both preferential accumulation of ANTs in cardiac mitochondria, and direct inhibition of cardiac topoisomerase 2 (Top2β) [48]. Other proposed mechanisms for ANT cardiotoxicity include dysregulation of calcium homeostasis via impairment of the cardiac ryanodine receptor and the sarco-/endoplasmic reticulum Ca2^+^ ATPase, and the impairment of mitochondrial dynamics (fusion, fission, and mitophagy). As a result, cardiomyocytes exposed to ANTs suffer from increased oxidative stress, inhibition of energy production, calcium overload, and reduced mitochondrial clearance.

Other anticancer drugs with cardiotoxic effects include the tyrosine kinase inhibitors imatinib [49,50], sunitinib [51], and trastuzumab [52]; while imatinib and sunitinib have been shown to exert a directly toxic effect on cardiac mitochondria via an increase in oxidative stress [51,53], trastuzumab has the capability to modify the expression of regulatory genes of mitochondrial function and DNA repair, leading to mitochondrial-induced apoptosis [54].

#### 3.2.4. Carbon Monoxide

Carbon monoxide (CO) is a toxic agent with a high affinity to hemoglobin. It has been linked to various phenotypes of CMP after acute exposure [55,56] and to angina, MI, arrhythmias, and heart failure [57]. Its toxic effect on the heart is thought to derive mainly from CO binding to cardiac myoglobin, impairing oxygen delivery to mitochondria; however, it is now clear that CO also has a direct effect on cardiac mitochondria via the inhibition of cytochrome c oxidase (and subsequently, impairment of the respiratory chain) and a decrease in glutathione levels [57].

#### 3.2.5. Other Drugs

The thiazolidinedione rosiglitazione was approved for use in type-2 diabetes mellitus patients in 1999 by the U.S. Food and Drug Administration (FDA), and in 2000 by the European Medicines Agency (EMA). However, after doubts arose about the cardiovascular safety of the drug, both agencies revised their approvals, with the EMA fully withdrawing it in 2010 [58,59]. Various hypotheses exist on the nature of rosiglitazone cardiotoxicity. In particular, it may induce oxidative stress-mediated acceleration in apoptosis [60], and there is in-vitro evidence that administration of rosiglitazone at supratherapeutic concentrations harms cardiac contractility and causes mitochondrial dysfunction through an increase in mitochondrial oxidative stress and direct impairment of mitochondrial bioenergetics [61].

Azidothymidine is an antiretroviral nucleoside analog approved for use in AIDS patients [62]. Long term use of azidothymidine is associated with the development of CMP, as azidothymidine may act as a stimulator of ROS production in mitochondria [63]. Interestingly, pre-treatment with resveratrol has shown a protective effect against cardiotoxicity by azidothymidine [64].

A comprehensive review of drugs eliciting mitochondrial dysfunction and subsequent cardiotoxicity has been extensively treated elsewhere [65].

### 3.3. Metabolic Cardiomyopathies: The Case for Diabetes and the Metabolic Syndrome

Metabolic CMPs can be defined as cases in which structural and functional alterations of the myocardium are secondary to inherited or acquired metabolic defects [66]. 

Inherited metabolic CMPs have been described elsewhere [66] and are beyond the scope of the present review. Acquired metabolic CMPs include diabetic and metabolic syndrome-related CMP.

Diabetic CMP has been defined as the presence of structural and functional alterations in the myocardium of patients with type-2 diabetes mellitus, which is independent of the conventional effect diabetes exerts on the vascular system and which does not recognize any other cause. It has been debated whether diabetic CMP exists [67], but current evidence supports the idea that specific myocardial alterations can be described in diabetes mellitus patients [68]. A proposed spectrum for diabetic cardiomyopathy ranges from diastolic dysfunction alone to overt left ventricular systolic disfunction [69].

Mitochondrial dysfunction plays a key role in the proposed pathogenesis for diabetic CMP. In particular, hyperglycemia, insulin resistance, and hyperinsulinemia would favor mitochondrial dysfunction by causing an increase in oxidative stress and calcium overload [68]. Evidence of reduced MCU protein levels in mouse models with diabetes [24] further strengthens this hypothesis, because it would lead to reduced mitochondrial calcium import and impaired energy production. In the experimental setting, cardiac insulin receptor knockout was demonstrated to promote progressive mitochondrial dysfunction and reduction in myocardial metabolic efficiency [70]. Moreover, plasma levels of long-chain acylcarnitines (LCACs), proposed biomarkers of mitochondrial bottlenecks, were found to be increased in the presence of insulin resistance [71]. Mitochondrial dysfunction may occur independently of hyperglycemia, justifying the need for a specific, diabetes-related CMP entity [72].

A possible role for mitochondria has also been postulated for cardiac outcomes in patients with metabolic syndrome and nonalcoholic fatty liver disease (NAFLD). Bile acids are established cardiotoxic agents capable of impairing ventricular function and have been associated with an increased risk of atrial fibrillation [73]. Indeed, the demonstration of bile acid receptor expression in the heart and the assertion that activation of such receptors could trigger MPTP opening in cardiac mitochondria [74] supports the idea that metabolic diseases have a direct impact on cardiac metabolism and disease.

### 3.4. Conduction Disorders

From a pathophysiological point of view, mitochondrial dysfunction may cause conduction disorders in many ways [75]. 

Firstly, a consistent and continuous production of ATP is needed for the conduction system to function properly. Since cardiomyocytes bear ATP-sensitive K^+^ channels, reduced ATP production may result in alterations of K^+^ flux through the sarcolemma. These channels are sensitive to oxidative stress as well, so that increasing levels of ROS production and impaired mitochondrial energetics may trigger K^+^ dispersion and a subsequent fall in the cardiomyocyte action potential, paving the way for the onset of conduction defects.

Secondly, the cardiac action potential is dependent on the mitochondrial membrane potential, which is strictly linked to ROS concentrations [76]; thus, an increase in mitochondrial ROS production would harm the stability of cardiac action potential, leading to conduction failure. This process is self-sustaining, since mitochondria exposed to ROS are triggered to release more ROS (ROS-induced ROS release, RIRR) [37]. 

Finally, myocellular calcium overload has been associated with arrhythmogenesis both in inherited and acquired diseases [77]. Conditions causing mitochondrial calcium overload may determine an increase in oxidative stress and MPTP opening, causing the uncoupling of oxidative phosphorylation and harming the mitochondrial membrane potential. This, in turn, may worsen the calcium burden on the sarcoplasmic reticulum and possibly promote atrial fibrillation [78] or other arrhythmias.

## 4. Mitochondrial Involvement in Heart Failure

The established mitochondrial impairment in myocardial infarction, ischemia/reperfusion injury, various phenotypes of cardiomyopathy, and arrhythmias pave the way for HF syndrome.

HF has been described as a condition in which the heart behaves like an “energy-starved engine” [79]. As such, energy deprivation in the myocardium is pivotal to the pathogenesis of HF. 

A clinical classification of HF distinguishes between HF with reduced ejection fraction (HFrEF) and HF with preserved ejection fraction (HFpEF). These conditions differ in pathophysiological and clinical terms, but there is evidence that both are linked to mitochondrial abnormalities.

Histological and energetic abnormalities of mitochondria in heart failure have long been known, with one of the first comprehensive reports being published in 1966 [80]. More recently, experimental models confirmed a significantly reduced mitochondrial size [81] and abnormal function [82] in dogs with HF; such findings have been replicated in humans [83]. 

Moreover, MR spectroscopy demonstrated that a reduced phosphocreatine-to-ATP (PCr/ATP) ratio, which indicates impaired mitochondrial function, is a predictor of mortality in patients with HFrEF [84]. A reduced PCr/ATP ratio has since been demonstrated also in HFpEF [85]. 

A metabolomics profiling study in a cohort of more than 500 HF patients showed that long-chain acylcarnitine (LCAC) levels are increased in patients with both HFrEF and HFpEF [86]. LCACs are transient intermediates of fatty acids oxidation; their accumulation in the plasma represents a marker of inefficient fatty acid oxidation, also due to mitochondrial enzyme defects [71]. Finally, LCAC may well represent a plasma biomarker for metabolic/mitochondrial dysfunction in patients with HF.

A role in the pathogenesis of HF has been proposed for mitochondrial complex I deficiency. Complex I is the largest component of the electron transport chain, including over forty subunits, several of which are subject to mutation [87]. Complex I deficiency by mutation is associated with mitochondrial diseases, cancer, neurodegenerative diseases including Parkinson’s disease and Alzheimer’s disease, and various phenotypes of CV disease, including decompensated hypertrophy, IRI, diabetic CMP, and HF [88].

In all cases, dysfunction of complex I causes an excess in ROS production and activation of cell death via opening of the MPTP. In mice models, complex I-deficient animals showed impaired systolic function and reduced cardiac output [89]. In a similar, more recent investigation, deficient mice were found to be more susceptible to chronic stress (i.e., pressure overload) with increased HF occurrence with respect to wild-type mice [90], but no alterations were reported under unstressed conditions. Interestingly, iron deficiency was shown to cause a reduction in complex I activity and left ventricle (LV) dysfunction in mice [91]; this could possibly explain HF onset in the setting of iron deficiency.

Finally, mitochondrial abnormalities in HF are not limited to ultrastructural changes and impairment of energetic metabolism; in fact, there is evidence that mitochondrial biogenesis is altered [92], that the protective role of mitophagy is lost [19], and that unbalanced mitochondrial dynamics does take place in HF, with an excess in mitochondrial fission and a reduction of mitochondrial fusion [13].

Notably, some authors have pointed out that mitochondrial dysfunction in HFpEF may go beyond the heart. In fact, abnormalities of mitochondria from skeletal muscles of HFpEF patients have been reported [93], which could explain why, in a 6-study meta-analysis of such patients, amelioration of cardiorespiratory fitness by training was not accompanied by significant changes in LV systolic or diastolic function [94]. As such, any therapeutic agent targeted on the mitochondrion in HF would likely produce its benefits by acting both on cardiac and noncardiac mitochondria.

## 5. Targeting the Mitochondrion in Heart Failure

Targeting mitochondrial function in HF may deliver a number of advantages. 

In the setting of HFrEF, the addition of mitochondrial-targeted therapy to current treatment strategies could benefit the patient by restoring the normal metabolic milieu, potentially reverting the decline in cardiac substrate usage capabilities. 

As for HFpEF, the heterogeneity of cardiac disease phenotypes has prevented significant therapeutic advancements [95,96,97,98], and no specific treatment is available to ameliorate morbidity and mortality in these patients [99]. However, mitochondrial dysfunction in both cardiac and noncardiac muscle cells has been reported in HFpEF [93], which could also explain exercise intolerance, a feature shared by virtually all patients [100]. 

We selected the most important therapeutic approaches investigated in the last decade for targeting both cardiac and noncardiac mitochondrial function in HF.

### 5.1. Mitochondrial Antioxidants: Elamipretide, mitoTEMPO, and mitoQ

Given the importance of oxidative stress in mitochondrial dysfunction and the pathogenesis of HF, various antioxidants have been investigated in HF treatment. Since general antioxidants fail to reach the mitochondrion, mitochondria-targeted antioxidants have been developed in recent years, including Elamipretide, MitoTEMPO, and mitoQ [88].

Elamipretide (formerly Bendavia, MTP-131, SS-31) is a mitochondrial-targeted tetrapeptide proposed for treatment of HF, which is capable of restoring mitochondrial function by interacting with cardiolipin on the inner mitochondrial membrane [101]. Cardiolipin is of pivotal importance to mitochondrial function, stabilizing mitochondrial energetics, biogenesis, and dynamics; a loss of cardiolipin expression has been documented in MI, IRI, heart failure, and the aging heart [102], probably due to increased peroxidation (ROS production). In dogs with advanced HF, chronic elamipretide administration led to ameliorated mitochondrial respiration and reduced ROS production [103]; such outcomes were confirmed in a study on explanted human mitochondria [104] after acute elamipretide exposure, suggesting elamipretide may exert some of its effects independently of cardiolipin. In the clinical setting, a single infusion of elamipretide improved ventricular volumes in patients with HFrEF with a dose–effect relationship [105]. However, results from the PROGRESS-HF study, which enrolled 71 patients in a double-blind, randomized controlled trial, showed no significant changes in LV volumes and systolic function among patients treated with 4-week daily administration of elamipretide [106].

As for HFpEF, two clinical trials exploring the efficacy of elamipretide have been reported as completed (NCT02814097, NCT02914665), yet no results are hitherto available.

MitoTEMPO is a mitochondria-targeted antioxidant that acts as a mimetic of superoxide dismutase; chronic administration of mitoTEMPO has been shown to prevent and reverse HF in animal models [107], and, interestingly, it was beneficial in mice with diabetic CMP [108] in whom it reduced the degree of left ventricular hypertrophy.

MitoQ, or mitoquinone, is a derivative of coenzyme Q capable of accumulating in the mitochondrial matrix [109]. In vivo studies on rats showed that mitoQ ameliorates oxidative stress and confers protection against cell death in the setting of IRI [110]. Further investigations showed improved mitochondrial energetics in rats with pressure-overload HF [111], but no significant effect of mitoQ on cardiac function. More recently, mitoQ was shown to improve mitochondrial network dynamics (concerning both intermitochondrial and mitochondrion-to-sarcoplasmic reticulum alignment) in a similar HF model [112].

### 5.2. Partial Adenosine A1 Receptor Agonists

Adenosine A1 receptor (A1R) agonists activate adenosine A1 receptors, mimicking the physiological role of adenosine. Adenosine has a well-established cardioprotective effect mainly derived from A1 receptor activation, which determines reduction of calcium overload and enhancement of energy substrates utilization [113]; such evidence suggests the possible use of A1R agonists for enhancement of mitochondrial function in HF.

A1R agonists are divided into two categories, namely full and partial agonists [114]. Use of full A1R agonists is limited by a plethora of possible adverse effects, including bradycardia, atrioventricular blocks, sedation, or undesired changes in blood pressure [114]. On the contrary, use of partial A1R agonists in the therapy of HF seems promising and is currently under investigation.

Among partial agonists, capadenoson and neladenoson have been evaluated in HF. 

Daily administration of oral capadenoson in dogs with HFrEF improved LV function after a 12-week period [115] without causing bradycardia or hypotension. In that same cohort, capadenoson was linked to improved myocardial energetics due to enhancement of mitochondrial biogenesis and oxidative capacity [116]. In a clinical study, capadenoson improved total exercise time in patients with stable angina [117]; however, in that same study, side effects such as dizziness and vertigo emerged that contraindicated further investigations. 

Neladenoson, a more selective A1R agonist, was then identified [114] and proposed as a prodrug (neladenoson bialanate hydrochloride) for solubility purposes. To date, two clinical trials have investigated the possible use of neladenoson, namely in patients with HFrEF [118] and in those with HFpEF [119], respectively. 

In the PANTHEON study of 462 patients with chronic HFrEF, 20 weeks of neladenoson administration did not demonstrate favorable effects on cardiac structure or function [118]; moreover, a dose-dependent decrease in renal function was observed. Similarly, for HFpEF, in the PANACHE study on 339 patients, no significant changes in exercise capacity could be observed after 20 weeks of treatment with neladenoson. 

### 5.3. SGLT2 Inhibitors

Sodium glucose cotransporters (SGLTs) are transporter proteins expressed to different extents in the nervous system, intestine, renal tubules, liver, lung, and heart [120]. 

Two main types of SGLTs are known, namely SGLT1 and SGLT2, and their biological and pathological roles have been investigated in the last 50 years [121]. Inhibition of SGLTs caught the attention of researchers in the 1980s, when it was demonstrated that administration of phlorizin (a natural, nonselective SGLT inhibitor) could induce glycosuria without hypoglycemia. Following studies showed that adverse effects of such administration—including diarrhea—were mainly due to SGLT1 inhibition in the intestine; subsequent analyses of the molecular structure of phlorizin and the SGLT receptor led to the development of SGLT2-selective inhibitors as antidiabetic drugs [122]. 

In more recent years, light has been shed on the possible cardioprotective effects of SGLT2 inhibitors, ultimately leading to the FDA approval of dapagliflozin for the treatment of HFrEF in 2020 [123]. 

To date, five major clinical trials have investigated the use of SGLT2 inhibitors in CV disease. Among these, EMPA-REG OUTCOME, the CANVAS Program, DECLARE-TIMI 58, and the CREDENCE trial did not specifically enroll patients with heart failure, but a meta-analysis of these studies demonstrated a protective effect of SGLT2 inhibitors with respect to CV death/MI/stroke (RR, 0.81; 95% CI, 0.70–0.94) and heart failure as well (RR, 0.61; 95% CI, 0.48–0.78) [124]. 

DAPA-HF, issued in 2019, was the first trial to specifically investigate SGLT2 inhibitors in diabetic patients with HFrEF. In this study, dapagliflozin was shown to protect from worsening HF (HR, 0.74; 95% CI, 0.65–0.85), to reduce CV death or HF-hospitalization (HR, 0.75; 95% CI, 0.65–0.85), and to confer mild protection against all-cause mortality (HR, 0.83; 95% CI, 0.71–0.97).

Even though there is mounting evidence of the clinical efficacy of SGLT2 inhibitors in HF, a comprehensive understanding of the pathophysiology underlying the cardioprotection of SGLT2 inhibitors is still lacking, also given that SGLT2 is not expressed in cardiomyocytes [120]. Hypotheses in this context range from a possible direct effect on cardiomyocytes to systemic effects on inflammation, apoptosis, and oxidative stress [125]. Whatever specific hypothesis is considered, a role for mitochondria emerges. 

In the failing heart, mitochondrial dysfunction determines the switch to anaerobic, glycolytic metabolism and a reduced ATP production capability of cardiomyocytes [79,126,127]. SGLT2 inhibitors can counterbalance this metabolic defection by increasing ketone bodies production via the lowering of glucose plasma levels. 

Moreover, some SGLT2 inhibitors have shown the capability to reduce inflammation, oxidative stress, and cardiac glucose uptake by inducing downregulation of SGLT1 expression in cardiomyocytes [125]; this framework of effects could reduce the burden of mitochondrial dysfunction. Furthermore, preclinical studies demonstrated that SGLT2 inhibitors block the functioning of the Na^+^/H^+^ exchanger [128], an integral membrane protein that is upregulated in heart failure and that was linked to arrhythmogenesis and increased ROS production [129,130,131]. As such, SGLT2 inhibitors may improve excitation–contraction coupling and reduce oxidative stress.

### 5.4. Mitochondria-Targeting Natural Compounds 

Given the importance of mitochondrial quality control mechanisms in ensuring normal cardiac function, and since the alteration of such mechanisms is commonly observed in the aging heart and HF syndromes, potential benefits of mitochondria-protecting molecules have been investigated [132,133]. Notably, anthocyanins, quinones, isothiocyanates, quercetin, urolithins, and spermidine yielded promising results. Anthocyanins are pholyphenoles commonly found in various fruits and vegetables, known for their antioxidant properties, and capable of increasing complex I function, which as previously discussed can be impaired in various contexts, including HF [132]. Studies on experimental IRI models showed that anthocyanins can prevent ischemia-induced apoptosis, ameliorate mitochondrial energetics, and reduce inflammation [134].

Quinones are natural metabolites found in various plants, fungi, and bacteria, with potent anti-inflammatory, antioxidant, and cardioprotective properties. As with anthocyanins, quinones were shown to be useful in IRI models, where they reduced infarct size and improved hemodynamics [135].

Isothiocyanates are mainly found in cruciferous vegetables. Although limited to the experimental setting, there is evidence that these molecules, and particularly sulforaphane, could improve cardiac function and remodeling by reducing mitochondrial oxidative stress and inflammation [136].

Quercetin is a ubiquitous flavonoid known for its favorable effects on the heart [132]. A recent systematic review and meta-analysis of 12 studies addressing the effects of quercetin on cardiac function in pressure overload and IRI models reported that use of quercetin is indeed associated with significant LV function improvement [137].

Urolithins are metabolites of pomegranates generated by the gut microbiota [133]. Urolithin A has been shown to prevent cardiac dysfunction in diabetic rats [138] and to suppress cardiac fibrosis in a model of diabetic CMP [139], possibly exerting these effects by activating mitophagy and thus increasing mitochondrial clearance. Moreover, a recent study found that urolithin B is effective in protecting against IRI, both in vivo and in vitro [140]. 

Spermidine is commonly present in rice bran, mushrooms, wheat germ, and other foods. It is considered an “anti-aging” food [141] with powerful effects on the heart. Spermidine was found to ameliorate cardiac autophagy, mitophagy, and mitochondrial respiration in rats, also reducing systemic blood pressure and delaying age-related diastolic function in a congestive HF model [142]. The proposed mechanism of spermidine-dependent cardioprotection is similar to that of resveratrol, a calorie-restricting mimetic [143] with anti-inflammatory and antioxidant effects.

## 6. Materials and Methods

The authors performed a computerized systematic review of the literature and searched Medline, the Clinical Trials Registry, the Cochrane Library, Web of Science, Google Scholar, ResearchGate, as well as reference lists of all identified articles and previous reviews and meta-analyses, from January 1966 through November 2020 for potentially relevant articles; ultimately, a selection of most relevant papers was finally included in the current review according to the authors’ opinions. 

## 7. Conclusions

Mitochondria play a key role in the pathogenesis of heart disease. Energy depletion, calcium overload, increased apoptosis, oxidative stress, and impaired mitochondrial dynamics are core components of cardiac dysfunction leading to cardiomyopathy, arrhythmogenesis, ischemia, and finally heart failure, Translational outcomes of mitochondrial targeted therapy are highly anticipated, and further investigations are needed to validate both natural compounds and medical substances in the real-world setting.

## Abbreviations

A1R	Adenosine A1 receptor
AIDS	Acquired immune deficiency syndrome
ANTs	Anthracyclines
ATP	Adenosine triphosphate
CANVAS	Canagliflozin Cardiovascular Assessment Study
CVD	Cardiovascular disease
CMP	Cardiomyopathy
CO	Carbon monoxide
CREDENCE	Canagliflozin and Renal Endpoints in Diabetes with Established Nephropathy Clinical Evaluation
DAPA-HF	Dapagliflozin in Patients with Heart Failure and Reduced Ejection Fraction
DECLARE-TIMI	Dapagliflozin Effect on Cardiovascular Events
DRP1	Dynamin-related protein 1
EMA	European Medicines Agency
EMPA-REG	Empagliflozin Cardiovascular Outcome Event Trial in Type 2 Diabetes Mellitus Patients
FDA	U.S. Food and Drug Administration
FIS1	Mitochondrial fission 1 protein
HF	Heart failure
HFpEF	Heart failure with preserved ejection fraction
HFrEF	Heart failure with reduced ejection fraction
IRI	Ischemia/reperfusion injury
LCAC	Long-chain acylcarnitine
LV	Left ventricle/left ventricular
MCU	Mitochondrial calcium uniporter
METH	Methamphetamine
MFN1	Mitofusin 1
MFN2	Mitofusin 2
MI	Myocardial infarction
MPTP	Mitochondrial permeability transition pore
NAFLD	Nonalcoholic fatty liver disease
NCLX	Li^+^-permeable Na^+^–Ca^2+^ exchanger
OPA1	Optic atrophy 1, mitochondrial dynamin like GTPase
OXPHOS	Oxidative phosphorylation
PGC-1α	Peroxisome proliferator-activated receptor gamma coactivator 1-alpha
RIRR	ROS-induced ROS release
ROS	Reactive oxygen species
SGLT	Sodium glucose cotransporter
SigmaR1	Sigma-1 receptor
SR	Sarcoplasmic reticulum
